# Real internal microstructure based key mechanism analysis on the micro-damage process of short fibre-reinforced composites

**DOI:** 10.1038/srep34761

**Published:** 2016-10-07

**Authors:** Xiaofang Hu, Jian Fang, Feng Xu, Bo Dong, Yu Xiao, Luobin Wang

**Affiliations:** 1 CAS Key Laboratory of Mechanical Behavior and Design of Materials, Department of Modern Mechanics, University of Science and Technology of China, Hefei 230026, China; 2 China Academy of Engineering Physics, Mianyang 621900, China

## Abstract

In this work, the underlying micro-damage mechanisms of randomly oriented short fibre-reinforced composites were revealed based on real internal microstructural characteristics obtained by high-resolution (0.7 μm/pixel) synchrotron radiation X-ray computed tomography (SR-CT). The special ‘pore dominant micro-damage processes’ were directly observed through SR-CT three-dimensional reconstructed images, which were different from the well-known ‘fibre breakage dominant failure mode’. The mechanisms of pore formation and pore evolution were further investigated on the basis of the microstructural parameters extracted from the SR-CT results. On one hand, the pore formation mechanism caused by shear stress concentration was proposed by combining the shear-lag model with the microstructural parameters obtained from the experiment, including the fibre length and orientation angle. On the other hand, the ‘fibre-end aggregation-induced pore connection’ mode of crack initiation was proposed through a composites model, which considered the parameters of real internal microstructure, including the critical value of the distance between neighbouring fibre ends and the number of neighbouring fibre ends. The study indicated that the shear stress concentration was significant in the region with a large number of neighbouring fibre ends, thus causing pore connection and crack initiation.

Randomly oriented short fibre-reinforced composites hold great potential for use as load-bearing materials because of the composites’ balanced property profile under multi-axial loading conditions^1^,^2^,^3^. However, these materials are not widely adopted as major force-bearing components in high-technology fields. One main reason is the lack of understanding on the underlying micro-damage mechanisms of randomly oriented short fibre-reinforced composites owing to the complexity of such composites’ internal microstructure. Unlike the simple microstructure in composites characterised by regular, aligned reinforcements, the randomly oriented fibre arrangement of randomly oriented short fibre-reinforced composites complicates the material’s internal microstructure and micro-damage mechanisms. Traditional two-dimensional experimental methods barely served as a driving force to reveal the underlying micro-damage mechanisms because such approaches cannot precisely characterize the relationship between real internal microstructure parameters and damage evolution. Ba Nghiep Nguyen and Mohammad A. Khaleel mentioned that ‘due to the complexity of their microstructure, damage in short-fibre composites was extremely difficult to assess numerically or experimentally’^4^. The prediction models developed over the last three decades were based only on simplistic assumptions of uncertain validity^5^,^6^,^7^,^8^,^9^. Therefore, investigating micro-damage process in short fibre-reinforced composites by considering real internal microstructure is an essential approach to uncover the underlying micro-mechanisms. The synchrotron radiation X-ray computed tomography (SR-CT) technique is an effective method for characterising real internal microstructure and has been used to study the damage evolution of unidirectional fibre-reinforced composites in a series of recent studies^10^,^11^,^12^,^13^,^14^. Given the complexity of the internal microstructure of randomly oriented short fibre-reinforced composites, increased spatial resolution and enhanced image quality in SR-CT experiments are necessary to successfully elucidate the underlying micro-damage mechanisms.

In this paper, the underlying micro-damage mechanisms of randomly oriented short fibre-reinforced composites were revealed, for the first time, on the basis of real internal microstructure characteristics obtained by high-resolution (0.7 μm/pixel) SR-CT experiments. The special ‘pore dominant micro-damage processes’ were directly observed in our experiment through SR-CT 3D reconstructed images, unlike the well-known ‘fibre-breakage dominant failure mode’. The corresponding micro-mechanisms of pore formation and pore evolution were further investigated by considering the influence of microstructure parameters extracted from the SR-CT results. Firstly, we proposed a pore formation mechanism caused by shear stress concentration by combining the shear-lag model with the fibre length and orientation angle. Through experimentation, we found that these factors were the two key microstructural parameters for pore formation. The value of shear stress at fibre ends, which caused pore formation, was determined by the fibre length and orientation angle. Secondly, we proposed the ‘fibre-end aggregation-induced pore connection’ mode of crack initiation through a composites model that demonstrated the significant influence of the microstructural parameters obtained from the experiment. Such microstructural parameters included the number *N*_*e*_ of neighbouring fibre ends and the critical value *l*_*e*_ of the distance between neighbouring fibre ends. The number *N*_*e*_indicated that the region with maximum number of neighbouring fibre ends was the weakest in the sample, thereby initiating sheet crack formation, as confirmed by the experimental results of two samples. The critical value *l*_*e*_ was determined by the properties of the composites, such as the interfacial shear strength in the short fibre-reinforced composite. This finding was also confirmed in our experiment by the decreased critical value of the composite after oxidation treatment, considering that oxidation treatment improved the interfacial shear strength.

## Results

### Pore formation and sheet crack initiation as fracture sources of the short-fibre composites

**Figure 1:**
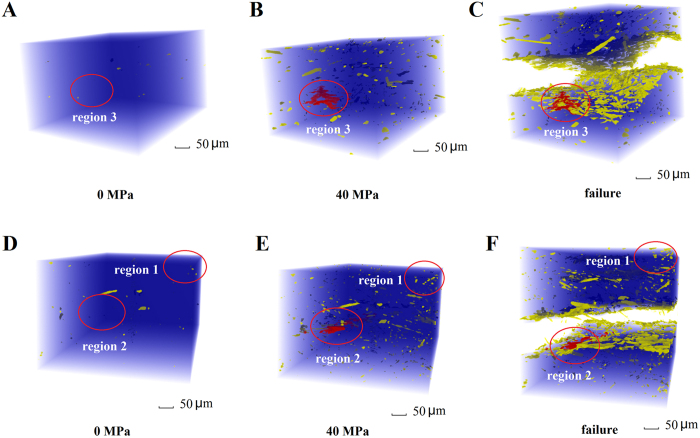
SR-CT 3D images of the microdefects in sample 1 (containing untreated short carbon fibres) under (**A**) 0 and (**B**) 40 MPa loads and (**C**) at sample failure and at sample 2 (containing short carbon fibres treated with oxidation) under (**D**) 0 and (**E**) 40 MPa loads and (**F**) at sample failure.

The micro-damage processes that include microdefect formation and evolution were the source of material fracture. For randomly oriented short fibre-reinforced composites, pore microdefects were observed directly by SR-CT. Two types of composites for the observation experiments were used: sample 1 contained a 15% fibre volume fraction of untreated short carbon fibres, whereas sample 2 contained a 15% fibre volume fraction of oxidation-treated short carbon fibres. Figure 1A–F separately show the SR-CT 3D morphology images of the pores in two types of samples under the loads 0 and 40 MPa and failure, which represent the initial microstructure, deformation stage and abrupt fracture, respectively. For clarity, these images display only the microdefects after removal of the epoxy resin and fibre signals by image extraction technology. Two obvious phenomena on pore formation and evolution are presented in Fig. 1. Firstly, large numbers of pores formed at some special region of the matrix in the deformation stage (Fig. 1B,E). Secondly, the evolutionary trend of these pores differed from one another. Most of the pores did not show significant changes after formation, as shown in region 1 of Fig. 1D–F, but the other part connected to a large sheet crack, which finally evolved into the fracture surface as shown in region 2 of Fig. 1A–C and region 3 of Fig. 1D–F.

The micro-damage processes in Fig. 1, including pore formation and sheet crack initiation, were the fracture sources in the randomly oriented short fibre-reinforced composites. The ‘pore dominant micro-damage processes’ observed in our experiment (Fig. 1) differed from that of the microstructure evolution of the fibre-reinforced composite in many previous studies^5^,^15^, such as the fibre-breakage dominant mechanism. To further elucidate the micro-damage mechanisms in our experiment, we analysed the influence of real internal microstructure on the micro-damage evolution by SR-CT.

### Mechanism of pore formation caused by shear stress concentration

**Figure 2:**
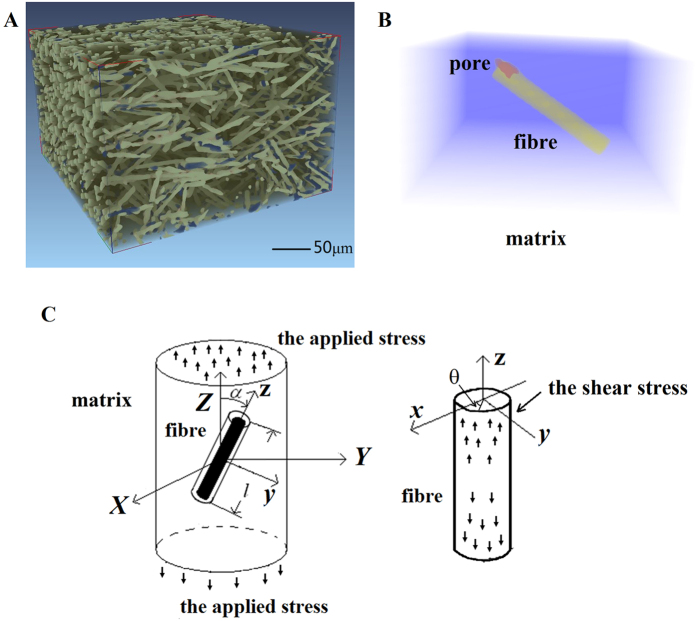
(**A**) Relative positional information of each pore and adjacent fibres at a load of 40 MPa. (**B**) Single-fibre element of the sample extracted to investigate the pore-forming process. (**C**) Shear-lag method in the fibre-reinforced composite.

To reveal the mechanisms of pore formation, we further extracted the fibre arrangement in the sample because such arrangement corresponds to the real internal microstructure of the short fibre-reinforced composites. The 3D images in Fig. 2A show the microstructure of sample 1 at a load of 40 MPa obtained by SR-CT. The fibres are marked in green, and the pores are highlighted in blue. Each single fibre and the respective surrounding matrix in sample 1 were extracted. One example was shown in Fig. 2B. Extraction of all fibres in sample 1 showed that all the pores were found in contact with a fibre end. The shear-lag method^16^ in fibre-reinforced composite was introduced (Fig. 2C) to analyse the influence of fibre ends on pore formation. The shear-lag method holds that when a fibre is introduced into the matrix, the shear stress of the fibre–matrix interface reaches its maximum at the fibre end. With the increase of the applied stress, the shear stress concentration at fibre end would be serious. When the shear stress at fibre end exceeded the interfacial bonding strength between fibre and matrix, nucleation of pore microdefect would occur at the two phase boundaries. Therefore, pore formation was caused by the shear stress concentration at the fibre end. However, given the extraction of all fibres in Fig. 2B, some fibres were not in contact with pores at their ends. Thus, the pore formation may be influenced by some microstructure parameters, such as fibre length and orientation, which was discussed in the following section on the basis of the SR-CT experimental data.

**Figure 3:**
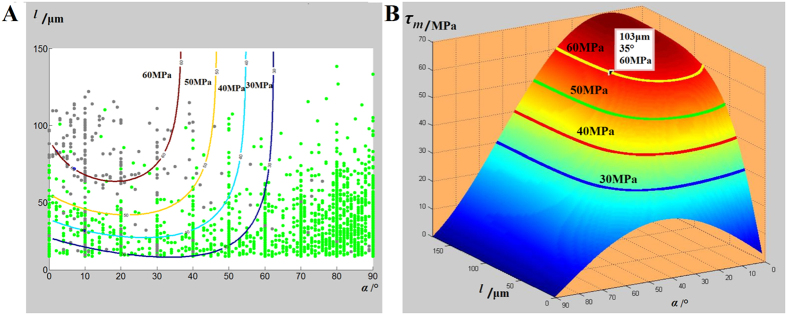
(**A**) Geometrical parameter of the fibres with and without pores at their ends. (**B**) Theoretical prediction of the max shear stress curve with different fibrous geometrical parameters.

Given the 3D images in Fig. 2B, we directly obtained the pore location, spatial attitude of single fibres and fibre length and orientation. The results indicated that only some fibres were in contact with pores at their end, whereas the interface between the other fibres and the matrix maintained the bonding well. In Fig. 3A, fibres with pores at their ends were plotted as grey solid dots, with orientation angle *α*, which was the angle between the fibre’s orientation and the applied stress direction on the horizontal axis, and fibre length *l* on the vertical axis. The other fibres were marked in green. In Fig. 3A, pore formation is shown mainly around the fibres at a small orientation angle and with a long fibre length (most of the fibres were less than 40° and more than 50 μm). These results could help reveal the mechanism of pore formation using mechanical models. For further study of the influence of fibre length *l* and orientation angle α, the general method of shear lag^16^ was adopted to calculate the shear stress. Thus, the shear stress *τ*_*m*_of the fibre direction at the load of 40 MPa was calculated based on the shear-lag theory using the following formulas:


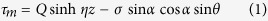


where 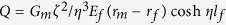, 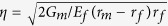, 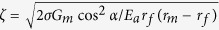 and 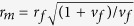 where *z* is the distance from the middle of fibre; σ is the applied stress (40 MPa); α is the fibre orientation angle; θ is the polar angle measured from the plane, which is normal to the fibre direction; *G*_*m*_is the shear modulus of the matrix material (1.01 GPa); *E*_*f*_ is the Young’s modulus of the carbon fibre (220 GPa); *r*_*f*_ is the radius of the fibre; *l*_*f*_is half of the length of the fibre; *r*_*m*_is the radius of the matrix sheath that surrounds the fibre. The value *r*_*m*_ was calculated through the value of *r*_*f*_ and *v*_*f*_ according to the equation 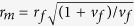; *E*_*a*_ is the Young’s modulus of the composite (4.54 GPa) which is obtained through uniaxial tension test. Five samples were prepared for the composites and the mean was obtained to eliminate the error; *v*_*f*_ is the fibre volume fraction of the short fibres (15%).

Given the above-mentioned formulas, the stress field that resulted in pore formation was obtained. In Eq. (1), the shear stress reached its maximum at the end of the fibre, which was consistent with the pore formation of the fibre ends observed in the experiment. Furthermore, the maximum shear stress changed dramatically with different fibre lengths *l* or orientation angles *α*. Figure 3B shows the change in the maximal shear stress value as a function of the fibre length *l* and orientation angle *α*. Four stress contour lines, which represent the stress states of 30, 40, 50 and 60 MPa, respectively, are highlighted with four different colours. We also compared the four contour lines with experimental results (Fig. 3A). Most of the fibres with pores at their ends extended above the red border (60 MPa). Thus, a critical value of shear stress potentially exists, such that the value determines whether the end of one fibre was in contact with the pore or without pore. This phenomenon indicated the validity of the shear stress concentration-induced pore formation.

Pore formation, which was observed to be in contact with fibre ends, was studied and considered to be caused by shear stress concentration. Furthermore, the influence of microstructural parameters on pore formation was investigated. Fibre length and orientation angle were found to be two key microstructural parameters of pore formation that determined the value of shear stress at fibre ends. Our results indicated that the pore-forming process should be studied by considering fibre orientation, rather than using the same direction between load and fibre orientation as adopted in some previous studies^17^,^18^,^19^,^20^.

### ‘Fibre-end aggregation-induced pore connection’ mode of crack initiation

**Figure 4:**
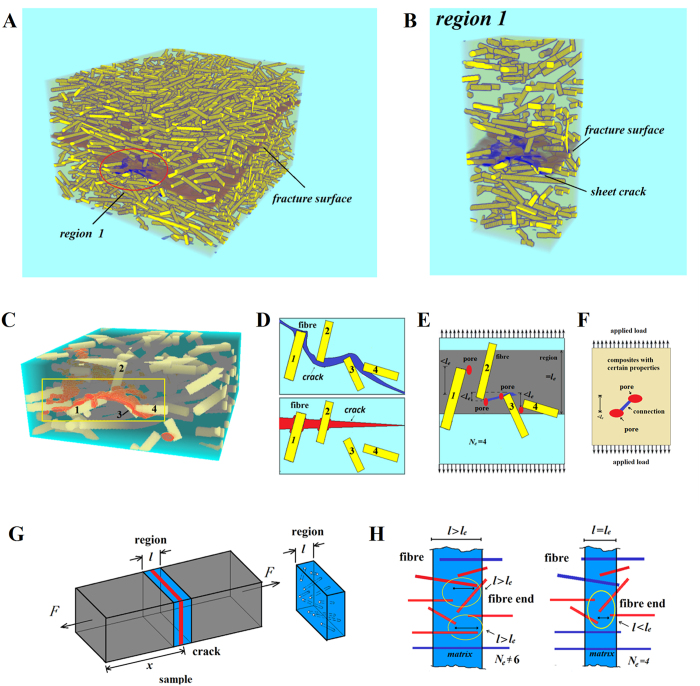
(**A**) Fibre arrangement with the ultimate fracture surface and the sheet crack caused by pore evolution. (**B,C**) Location of the sheet crack relative to the surrounding fibre ends. (**D**) Two different cracking behaviours in the fibre-reinforced composites, namely, the ‘fibre-end aggregation-induced pore connection’ mode and the ‘fibre-bridging crack’ mode. (**E**) Simplified model based on the actual arrangement of four fibres. (**F**) Critical value l_e_ in the composite with certain properties and applied load. (**G**) Region with maximum number N_e_ of neighbouring fibre ends and the position of crack initiation. (**H**) Region with different widths l and the distance between neighbouring fibre ends (marked in red) in the region.

Pore evolution caused the sheet crack initiation in Fig. 1B,E. To expose the sheet crack initiation mechanisms in the randomly oriented short fibre-reinforced composites, we investigated the microstructural features surrounding the sheet crack because these features reflect the process of crack initiation. By using SR-CT, we observed the sheet crack, the fibre arrangement around the sheet crack and the ultimate fracture surface (marked blue, yellow and red, respectively, in Fig. 4A). In region 1, the microstructural feature involving a sheet crack surrounded by a large number of fibre ends is apparent. Region 1 in Fig. 4A was magnified (Fig. 4B,C) to further obtain the location of the sheet crack relative to the surrounding fibre ends. A special crack path amongst fibres is observed in Fig. 4C, which could be considered as the connection of many neighbouring fibre ends (shown with blue bricks in Fig. 4D). This crack path was unlike from the widely known ‘fibre-bridging crack’ mode^21^,^22^,^23^,^24^,^25^ (shown with red bricks in Fig. 4D). In the traditional ‘fibre-bridging crack’ mode (red bricks in Fig. 4D), the crack propagated through the broken fibre-matrix interface, and the fibre bridged the top and bottom surfaces of the crack to hinder crack propagation. Considering the discussion in the above section, the special crack path was considered to be caused by pore formation at the area with many fibre ends and the connection of these pores. We called such process the ‘fibre-end aggregation-induced pore connection’ mode of crack initiation. The influence of the real microstructure feature in short fibre composites on this mode was discussed and further validation of this mode was conducted based on SR-CT experiment results, as described in the following section.

The influence of the real microstructural feature in the short fibre composites on the ‘fibre-end aggregation-induced pore connection’ mode was further analysed, and two key microstructural parameters *l*_*e*_ and *N*_*e*_were proposed. The composites model in Fig. 4E was adopted on the basis of the actual arrangement of four fibres in Fig. 4C. As mentioned in the above section, the pore formation at fibre ends (marked in red in Fig. 4E) was caused by the shear stress concentration. When the distance between the neighbouring pores along the direction of the applied stress was less than a critical value, pores could be connected under applied load, as shown in Fig. 4E. For the composite with certain properties and applied load in Fig. 4F, the critical value was certain and denoted by *l*_*e*_. Consequently, the region with maximum number *N*_*e*_of neighbouring fibre ends was weakest in the sample, and pores at fibre ends could be connected and cause sheet crack initiation (Fig. 4E,G). However, the critical value *l*_*e*_ in the two samples of our experiment was unknown. To determine the value of *l*_*e*_, the region with different widths *l* was removed from the real sample in Fig. 4H. When the width *l* was the critical value *l*_*e*_, the distance between neighbouring fibre ends (marked in red) in the region was less than *l*_*e*_ (Fig. 4H). Furthermore, the region with the maximum value of neighbouring fibre ends was consistent with the position of the sheet crack in our experiments. Basing on the SR-CT experimental results, we determined the critical value *l*_*e*_ in two samples. This result may serve as critical experimental evidence supporting the ‘fibre end aggregation-induced pore connection’ mode.

**Figure 5:**
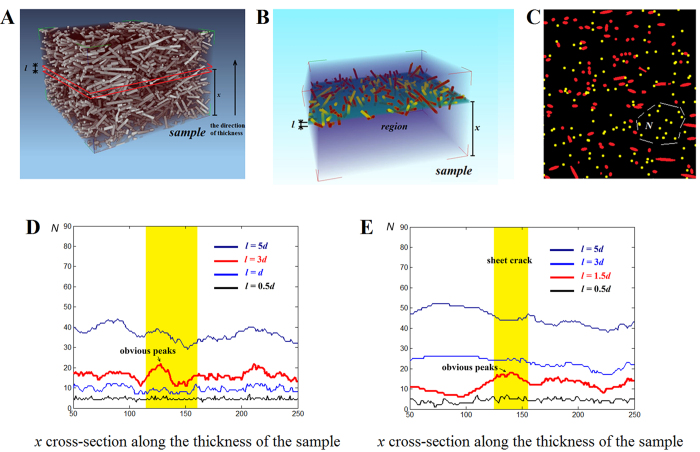
(**A**) Randomly oriented short fibre arrangement in the sample. (**B**) Region of given width l and quantitative analysis of fibre ends in the region. Fibre ends were marked in yellow, and other fibres were marked in red. (**C**) Vertical projection of the region. The maximum number of neighbouring yellow fibre ends (highlighted with white dash lines) is the value of N in the region. The N–x curve for (**D**) sample 1 (containing untreated short carbon fibres) with widths 0.5, 1, 3 and 5d, respectively, and (**E**) sample 2 (containing short carbon fibres treated with oxidation) with widths 0.5, 1.5, 3 and 5d, respectively.

Given the SR-CT experimental results, we removed the region with different widths in Fig. 4G from any position of the real sample to determine the critical value *l*_*e*_. As shown in Fig. 5A, the region with fixed width *nd* sweeps across the whole sample along the sample thickness, where *n* is a constant parameter and *d* is the fibre diameter. Then, in each region, fibre ends are marked in yellow in Fig. 5B, and the maximum number *N* of neighbouring fibre ends was obtained on the basis of the largest yellow-colour-connected region in Fig. 5C. Finally, in Fig. 5D–E, the relationship between fibre ends of number *N* and the height *x* of the region with fixed width *nd* were plotted as *N–x* curves, where *x* was the height of the cross-section along the thickness of the sample. A series of *N–x* curves was obtained when the region width *nd* decreased (Fig. 5D,E). This series was then compared with the fibre end aggregation-induced pore connection mode mentioned in Fig. 4G,H. According to the fibre-end aggregation-induced pore connection mode, the region with the maximum number of neighbouring fibre ends should be consistent with the position of the sheet crack until *nd* was reduced to the critical value *l*_*e*_. This finding can be directly verified from the two samples in Fig. 5: when the width *nd* of the *N–x* curve was reduced to the suitable width *l* (3d for sample 1 and 1.5d for sample 2), the obvious peak in the *N–x* curve was found to be consistent with the position of the sheet crack (highlighted in yellow) (Fig. 5D,E). Repeated observations in two samples of the same trends suggested that this phenomenon was a feature of randomly oriented short-fibre composites and was not induced by random defects. This result was critical experimental evidence supporting the fibre end aggregation-induced pore connection mode.

The region in Fig. 5B was similar to the concept of ‘critical zone’^6^,^26^ which was used to predict the strength of short fibre composites. This concept is developed in the present paper to reveal the underlying micro-damage mechanisms of randomly oriented short fibre-reinforced composites by introducing the two key microstructural parameters *l*_*e*_ and *N*_*e*_of the real internal microstructure.

**Figure 6:**
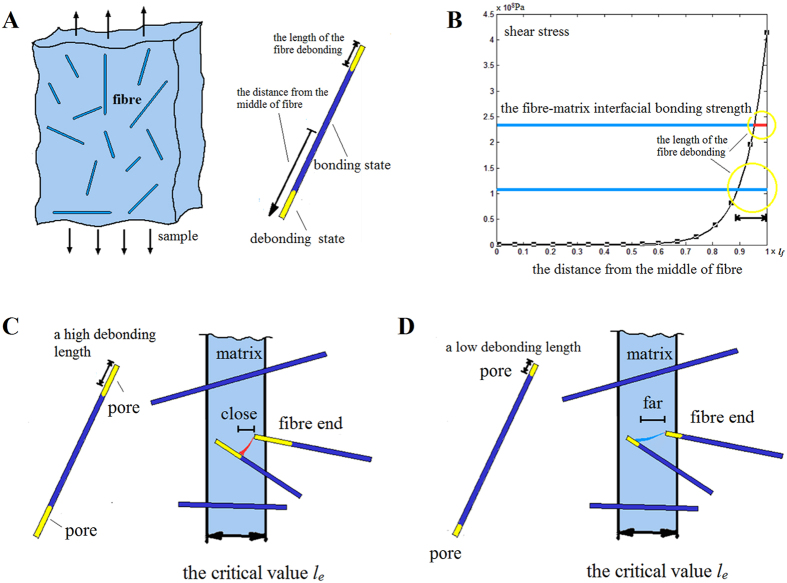
(**A**) Two types of fibre-matrix interface bonding states under loading. (**B**) Length of the fibre debonding section obtained from the interfacial bonding strength and the shear stress distribution along the fibre. Connection of microdefects in composites with (**C**) high and (**D**) low fibre debonding lengths.

Given the results in Fig. 5D,E, the critical values *l*_*e*_ in two samples (3d for sample 1 and 1.5d for sample 2 by the suitable width of the region) were different. This phenomenon could support the ‘fibre-end aggregation-induced pore connection’ mode through the following analysis. In Fig. 4F, the critical value *l*_*e*_ is considered certain in the sample with certain properties and applied load. Considering that the oxidation treatment can significantly increase the fibre-matrix interfacial bonding strength^27^,^28^,^29^,^30^,^31^,^32^, the oxidation treatment may affect the critical value *l*_*e*_. A fibre was debonded and pores formed because the shear stress exceeded the interfacial bonding strength. Therefore, when composites are under loading, fibre interface bonding states are divided into two types: debonding and bonding states (highlighted in yellow and blue, respectively, in Fig. 6A). On the basis of the interfacial bonding strength (blue line in Fig. 6B) and the shear stress distribution along the fibre obtained from Eq. (1) (black curve in Fig. 6B), we determined the length of the fibre debonding section (highlighted with yellow circles). The length of the fibre debonding increased as the interfacial bonding strength decreased (Fig. 6B). Meanwhile, the pore evolution in the composites with high and low fibre debonding lengths is presented in Fig. 6C,D. In short-fibre composites, the relative position between most neighbouring fibres was similar to the actual arrangement of four fibres in Fig. 4C; thus, the fibre arrangement in Fig. 6C,D was used. The position between the red pores in Fig. 6C was extremely close to being linked together, whereas the blue ones in Fig. 6D were far from each other. To ensure that the pores linked with each other, the critical value *l*_*e*_ in Fig. 6D should be smaller than that in Fig. 6C. Therefore, oxidation treatment improved the interfacial shear strength. Consequently, the length of the fibre debonding and the critical value *l*_*e*_ were reduced. This result was also a critical experimental evidence supporting the ‘fibre-end aggregation-induced pore connection’ mode.

The sheet crack initiation, which was observed to correlate with fibre-end aggregation, was studied, and the ‘fibre-end aggregation-induced pore connection’ mode of crack initiation was proposed. Given the real microstructural features around the sheet crack, the influence of microstructural parameters on the crack initiation mode was further investigated. The crack initiation was found to be controlled by microstructure parameters, including the number *N*_*e*_ of neighbouring fibre ends and the critical value *l*_*e*_ of the distance between neighbouring fibre ends. The value *N*_*e*_indicated that the region with the maximum number of neighbouring fibre ends was the weakest in the sample, thereby initiating sheet crack formation, as confirmed in the two samples of our experiment. The critical value *l*_*e*_was reduced in the short fibre-reinforced composite with low interfacial shear strength, which was consistent with the smaller critical value of composite subjected to oxidation treatment. Our result indicated that the crack initiation in short-fibre composites must be studied by considering the microstructural characteristics of fibre ends, which were difficult to be characterised by traditional two-dimensional experimental methods.

## Discussion

The underlying micro-damage mechanisms in randomly oriented short-fibre composites were revealed by precise SR-CT experimental characterisation of the relation between real internal microstructural characteristics and micro-damage evolution. The special ‘pore dominant micro-damage processes’ observed in our experiments were different from the well-known ‘fibre-breakage dominant failure mode’. These processes were shown to correlate to the special microstructural features in the composites, such as fibre length, orientation angle and fibre arrangement. By combining these microstructural features with mechanical models, further analysis was conducted, and the underlying micro-mechanisms were elucidated.

### The pore formation mechanisms caused by shear stress concentration were proposed on the basis of the special contact relationship between pore formation and fibre ends obtained from the SR-CT experiment

Two key microstructural parameters obtained from the experiments, namely, fibre length and orientation angle, were combined with the shear-lag model to further analyse the mechanisms of pore formation. By determining the value of shear stress at the fibre ends, we found that the fibre length and orientation angle could affect the pore formation. This view deviated from the damage micro-mechanisms of fibre-reinforced composites in some previous studies^17^,^18^,^19^,^20^, which considered that pore formation is caused by excess tensile stress in the form of fibre breakage.

### The ‘fibre-end aggregation-induced pore connection’ mode of crack initiation was proposed based on the special fibre-end aggregation features of the real internal microstructure surrounding the sheet crack

Two key microstructural parameters, particularly, the distance between neighbouring fibre ends *l*_*e*_ and the number of neighbouring fibre ends *N*_*e*_, were introduced in the proposed mode to consider the influence of the real microstructure in randomly oriented short fibre-reinforced composites. The value *N*_*e*_indicated that the region with maximum number of neighbouring fibre ends was the weakest area in the sample, causing the sheet crack initiation, as confirmed in our two samples. The critical value *l*_*e*_ was determined by the properties of the composites, a finding consistent with the smaller value *l*_*e*_ of the sample subjected to oxidation treatment.

## Method

Two types of composites for the observation experiments were prepared: sample 1 had a tensile strength of 97 MPa and contained a 15% fibre volume fraction of untreated short carbon fibres, whereas sample 2 had a tensile strength of 107 MPa and contained a 15% fibre volume fraction of oxidation-treated short carbon fibres. The composites were prepared by the three-roller method. Epoxy resin CYD-128, which is a low viscosity (62.5 Pa*s at 40 °C) diglycidyl ether of bisphenol-A type resin, and a hardener (2-ethylic-4-methyl imidazole, EMMZ) used as the matrix were supplied by the Sinopec Group. Epoxy resin CYD-128 was supplied by the Sinopec Group, and the short carbon fibres were supplied by Shanghai Carbon Factory; the short carbon fibres had an average diameter of 7 μm and an average length/diameter ratio of approximately 20. For the oxidation treatment, carbon fibres were oxidized in a furnace at 550 °C for 1 h, under the condition of constantly pumping air into the furnace^28^. For the SR-CT experiments, the CCD camera field of view was approximately 1 × 1 mm^2^ in this experiment. Therefore, the sample size was necessarily made correspondingly small. The dimensions of sample 1 were approximately 0.39 × 0.36 × 10 mm^3^ and the dimensions of sample 2 were approximately 0.56 × 0.34 × 10 mm^3^.

Test were carried out at the BL13W1 beam line in the Shanghai synchrotron radiation facility, and the samples were scanned prior to loading and then at incrementally increasing stress levels that ranged from 0 N to the point of final failure. Because the SCF/EP composite material was brittle, the last several stress levels were close to the tensile strength of the composite, to approach the complete deformation process. The optics used in this study provided an isotropic voxel resolution of 0.7 μm, with the sample-detector distance set to 14 cm and a beam energy of 15 keV. Seven hundred twenty radiographs were taken at regular increments over 180° of rotation, each with an exposure time of 1 s. 3D volumes were analyzed using the commercial package VG Studio Max v2.0, and features of interest were identified and segmented. Further details of the imaging process are provided in Supplementary Information.

## Additional Information

**How to cite this article**: Hu, X. *et al*. Real internal microstructure based key mechanism analysis on the micro-damage process of short fibre-reinforced composites. *Sci. Rep.* **6**, 34761; doi: 10.1038/srep34761 (2016).

## Supplementary Material

Supplementary Information
