# Supplementary material for: Real internal microstructure based key mechanism analysis on the micro-damage process of short fibre-reinforced composites
Source: Sci Rep. 2016 Oct 7;6:34761. doi: 10.1038/srep34761 (PMC5054434; doi:10.1038/srep34761)
Supplement: Supplementary Information [file srep34761-s1.pdf]

Supporting information

Real internal microstructure based key mechanism analysis on the micro-damage process of short fibre-reinforced composites

Xiaofang Hu[1], Jian Fang[1], Feng Xu[1,*], Bo Dong[1], Yu Xiao[1], Luobin Wang [2]

[1]*CAS Key Laboratory of Mechanical Behavior and Design of Materials, Department of Modern Mechanics, University of Science and Technology of China, Hefei 230026, China*

[2]*China Academy of Engineering Physics, Mianyang 621900, China*

A special tensile testing device (Fig. S1) was used to incrementally load sample to failure in situ with a displacement accuracy of 1 m and a force measurement accuracy of 0.1 mN. Test were carried out at the BL13W1 beamline in the Shanghai synchrotron radiation facility and the sample were scanned prior to loading and then at incrementally increasing stress levels ranging from 0N to the point of final failure. The optics used in this study provided an isotropic voxel resolution of 0.7 m, with the sample-detector distance being set to 14 mm and a beam energy of 15 keV. Because the tensile testing device blocks X-ray transmission during the rotation in an interval of about 50, the actual number of usable images is about 500 via a 2048×2048 detector system. Therefore, a reconstruction method for incomplete projection data was used to obtain the cross section images in this study and we called the reconstruction method “Filtered iteration reconstruction method”. This algorithm was developed by combining the Filtered Back Projection reconstruction method and iteration reconstruction method. First, the Filtered Back Projection reconstruction method was used based on original projection data. Then the results coming from

Filtered Back Projection reconstruction method was considered as the initial solution of iteration reconstruction method, which was used to obtain the cross section images. Further details of the reconstruction method are provided in Min WG et al. [1].

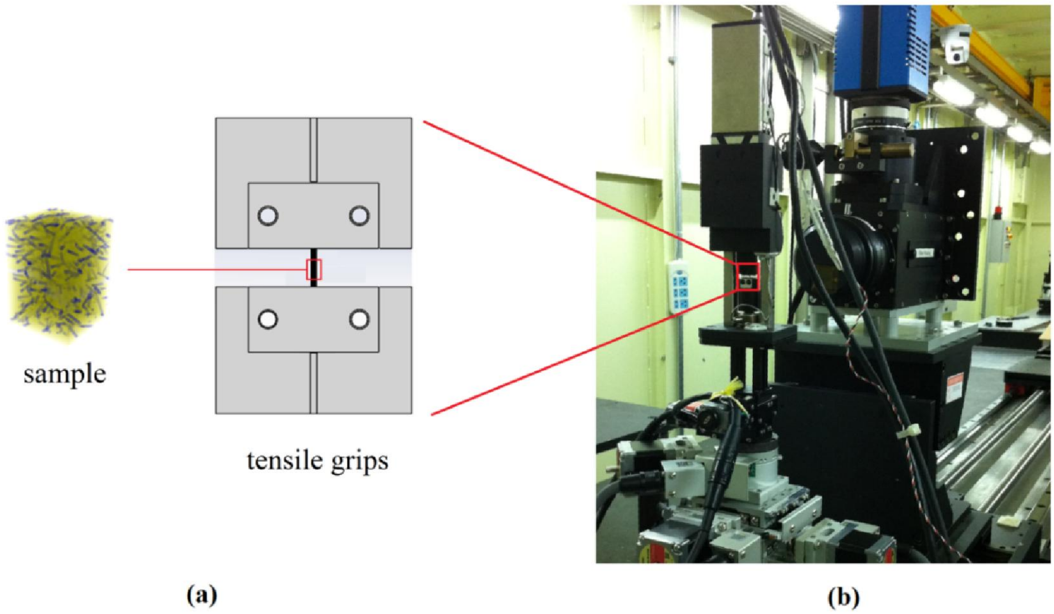

Figure S1. (a) the smaller loaded sample obtained for microtomography, (b) the special tensile testing device

3-D volumes were analyzed using the commercial package VG studio Max v2.0 and image edge extraction technologies were used to analyze microdefects, fractures and the geometric parameter of the fibres. Because of the different densities of the materials, the intensity of fibre, matrix and microdefect in the tomography were different, whereby the fibres and microdefects could be identified. For the geometric parameter of the fibres, the area of the fibres was recognized by adaptive threshold segmentation algorithms. A tool combining the 3-D watershed algorithm and region-merging technology was used for the segmentation of each fibre as shown in Fig. S2a-b. Based on the characteristics of the fibre, the extraction

of the fibre's parameters could be transformed to obtain the minimum circumscribed cylinder of a three-dimensional solid. Then, each fibre was divided into a cylindrical region and its geometric parameters could be calculated from the circumscribed cylinder as shown in Fig. S2c. For the microdefects and sheet crack, they could be individually tracked in the sample under different loads by the different densities of the materials in the tomography. Then the real microstructural features of fibre arrangement around the microdefects and sheet crack were obtained to analysis the micro-damage mechanisms as shown in Fig. S3.

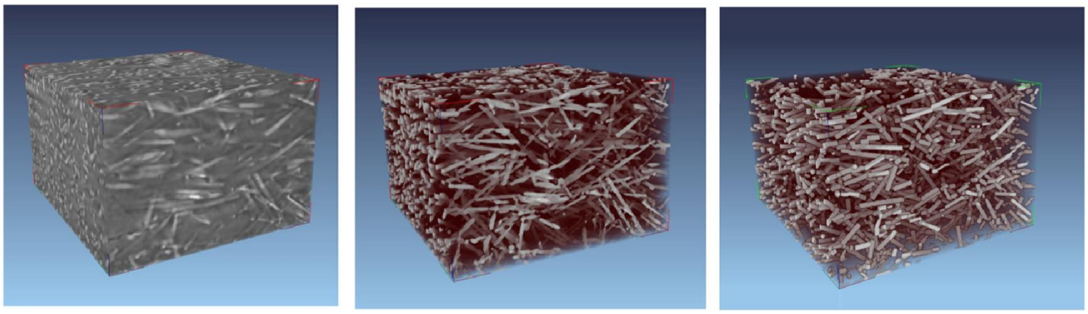

(a) (b) (c)

Figure S2. (a) SR-CT 3-D images of Sample using VG studio Max v2.0, (b) split image by adopting threshold segmentation, (c) replace each fiber into the minimum circumscribed cylinder of three-dimensional solid

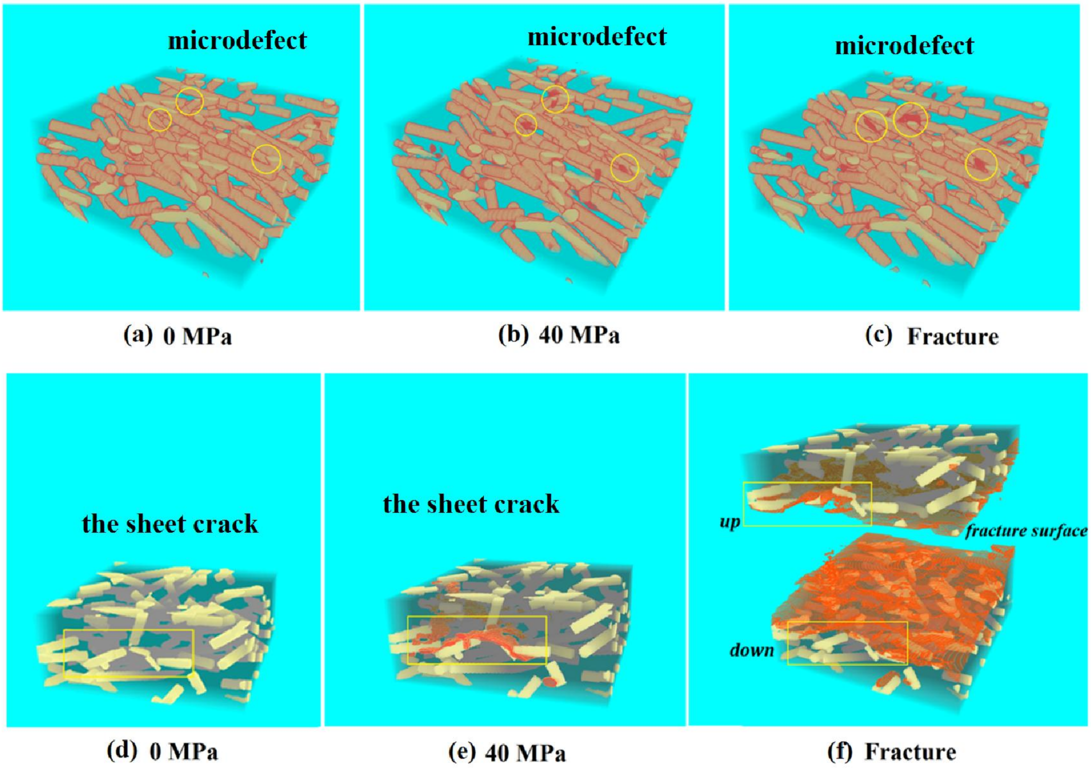

Figure S3. The real microstructural features of fibre arrangement around microdefects under (a) a 0 MPa load, (b) a 40 MPa load, and (c) at sample failure and the sheet crack under (d) a 0 MPa load, (e) a 40 MPa load, and (f) at sample failure.

Based on the microstructural features of fibre arrangement in the sample, the number *N* of neighbouring fibre ends in the manuscript can be obtained by the image processing method was as follows. 1) In Fig. S4a, a region was taken out from one position of the real sample based on a pair of planes separated by a given width *l*, where *l* was the width of the region and *x* was the height of the region along the thickness of the sample. 2) As mentioned in Fig. S2, each fibre in the sample was recognized and its geometric parameters including the centroid position, fibre length and fibre orientation was obtained. Then, each fibre of the sample was analyzed to verify whether it extended into the region in Fig. S4a and fibres which extended into the region were recognized in Fig. S4b. 3) Fibres which partially or totally extended

into the region were divided into two types in Fig. S4c. As show in Fig. S4c, fibres which crossed the region were marked in grey, and fibres whose ends were within the region were marked in blue. It is evident in Fig. S4c that the yellow fibre represents the fibre end in the region and the maximum number of neighbouring yellow fibre (highlighted with a red circle in Fig. S4c) is the value of N in the region. 4) In Fig. S4d, the sample consisted of many regions, and in each region with a different height x along the thickness of the sample, the values of N were calculated. Then, the relationship between N and x could be plotted as an N-x curve. By taking out different widths of the critical zone, different N-x curves could be obtained as shown in Fig. S4d. The width of the region was denoted by nd, where n is a constant parameter and d is the diameter of the fibre.

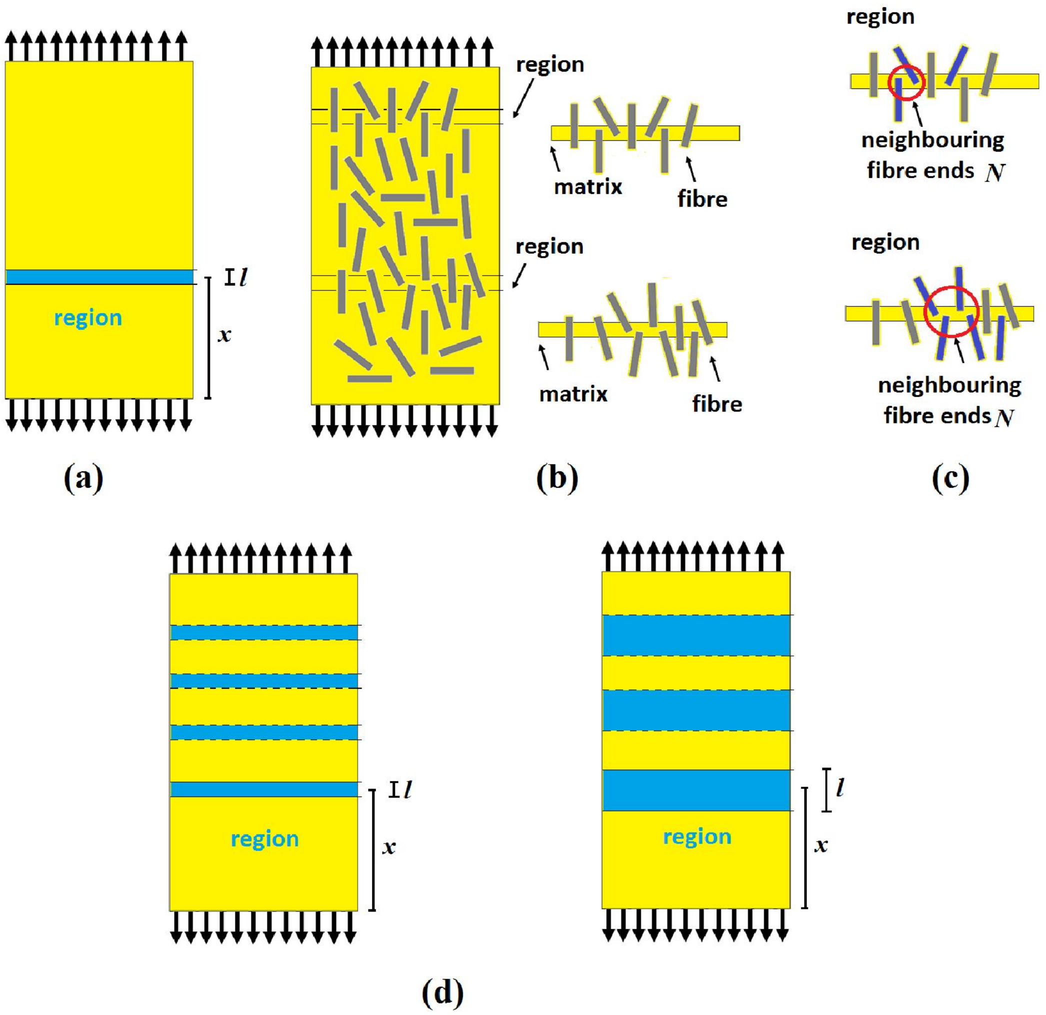

Figure S4. (a) A region of given width *l*. (b) The randomly oriented short fibre arrangement in the sample and quantitative analysis of fibre ends in the region. (c) Fibre ends were marked in yellow, and other fibres were marked in red (d) The *N*-*x* curve for sample with different widths.

1. WANG, M. & HU, X. F. Research on the incomplete projection data based on synchrotron radiation computed tomography technology. *Opt. Tech.* **6**, 035 (2006).
